# The risk of violating the posterior malleolar fracture when nailing the ipsilateral concomitant spiral distal tibial fracture

**DOI:** 10.1186/s12891-018-1994-x

**Published:** 2018-04-19

**Authors:** Yu Zhang, Xiaodong Qin, Lijun Song, Xiang Li

**Affiliations:** Department of Trauma, the First Affiliated Hospital of Nanjing Medical University and Jiangsu Province Hospital, 300 Guangzhou Road, Nanjing, 210029 China

**Keywords:** Ankle fracture, Tibial fracture, Morphological measurement, Intramedullary nail

## Abstract

**Background:**

For a distal tibial spiral fracture combined with a non-displaced posterior malleolar fragment (PMF), we proposed a hypothesis that the treating surgeon could assess the size of the PMF to determine the need for stabilizing that structure first before rodding the tibia.

**Materials and methods:**

Fifty 3-D models (22 females) of combined distal tibial and posterior malleolar fractures from one trauma center were reconstructed. In each case, a virtual tibial intramedullary nail (vIM nail) with three distal anteroposterior (AP) locking screws (S_13_, S_15_ and S_37_, the number indicating the distance from the screw to the nail tip) were inserted into the center of the tibial canal and ended on top of the distal tibial physeal scar. Contact between the screws and the PMF was defined as causing PMF displacement. The relationship between PMF secondary displacement and traumatic anatomic factors (the fragment area and height of the PMF) was explored. Then, the parameters were justified by analyzing intraoperative radiographs of 35 cases treated by nail with single locking screw (S_15_) design.

**Results:**

In the analog experiment, multiple logistic regression analysis revealed that the height of the PMF could confidently predict the risk of fragment displacement (S_13_: odds ratio [OR] 1.18, 95% confidence interval [CI] 1.06–1.32; S_15_: OR 1.15, 95% CI 1.05–1.27). Regarding the height of the PMF, the receiver operating characteristic established a cut-off value of 31.2 mm for preliminary fixation of the fragment with 88.89% sensitivity and 88.89% specificity. In the operation group the nail stopped on the top of distal tibial physeal scar, no PMF secondary displacement occurred when the PMF height was less than 31.2 mm. However, the incidence of secondary displacement was 93.33% when the height of the PMF exceeded 31.2 mm.

**Conclusion:**

When the distal tibial physeal scare was set as the limit of nail insertion depth, the height of the PMF could be used as a reliable reference predicting the risk of PMF secondary displacement caused by distal anteroposterior locking screw.

## Background

The combination of tibial shaft fracture and posterior malleolar disruption was first reported by Lauge-Hansen in 1946 [1]. Later, other authors recognized that a posterior malleolar fragment (PMF) could predominantly co-occur with a distal tibial spiral fracture [2, 3]. In 1988, Bostman further discussed this special lower limb injury and recommended additional CT scanning of the ankle for distal tibial spiral fractures [4]. Since then, the understanding of this special limb injury has been continuously increasing.

For tibial shaft spiral fractures, either proximal or distal, both a plate and an intramedullary nail can be utilized. However, the existence of the PMF and the “connection line” complicate the fixation selection since they change a tibial shaft fracture from a simple to a more complex intra-articular fracture [5]. Although the multiple distal locking option of the latest nail design greatly improves the holding strength of the short distal tibia segment and is compatible with a soft envelope, there is always a major concern about secondary displacement of an initial non-displaced PMF, especially those involving a substantial articular surface area, when inserting an IM nail or distal AP locking screws. The suggestion of indiscriminate PMF fixation with cannulated screws before nailing was made without sufficient supporting evidence and is not an effective solution [6]. This protocol might irritate the adjacent tendon (especially the hallux flexor longus from the posteroanterior screw) and ligaments, cause percutaneous nerve entrapment [7], exaggerate excessive patient radiation exposure and incur extra financial cost. Guo’s study also suggested that PMF fixation failed to improve functional outcomes when there was a moderate size PMF combined with a tibial spiral fracture [8]. As the treatment benefit should be balanced against the potential complications and costs, we believe it is wise to tailor the operation protocol for every case.

It could be deduced that when a smaller-sized PMF correlates with a lower risk of nail-induced secondary displacement of the fragment, this indicates that when the PMF size is small enough, the necessity of additional cannulated screw fixation of the fragment can be eliminated if the nail is inserted in the proper position. In this group of patients, a properly inserted IM nail could provide sufficient fixation without disrupting the non-displaced PMF. However, the proper threshold for the fragment size and a reliable anatomic reference for proper nail insertion depth have not been established. This paper investigated the factors influencing the intraoperative secondary displacement of PMFs caused by nail insertion and distal AP screw locking. By conducting radiographic measurements and simulating vIM nail fixation on a reconstructed 3-D model, the core morphological feature of the PMF predicting fragment violation from the nail and AP locking screw tips and its threshold could be determined. Then, an anatomical landmark that serves as a reliable reference for the proper depth of nail insertion could also be established. These results were validated in the same group of patients.

## Methods

### Patient and fracture evaluation

This is a retrospective study with ethically approved by the institutional review board of our Hospital (No. 2017-SR-121). From June 2011 to February 2017, 765 tibial fracture cases were retrieved from the orthopedic database in a territorial trauma center. The inclusion criteria were a spiral fracture at the distal third of the tibia combined with a posterior malleolar fracture confirmed by thin-slice CT scan (GE Light-Speed CT; Waukesha, WI, USA). Pathological fractures, skeletally immature patients, fractures without CT scan or with PMF contour obscured by an indistinct fracture line and severely comminuted tibial fractures with direct involvement of ankle joints were excluded from the study. Fifty-five patients (22 females) fulfilled the inclusion criteria and were included in the study. All subjects provided informed consent in written to take part in the study. Baseline characteristics of the patients were collected.

**Fig. 1:**
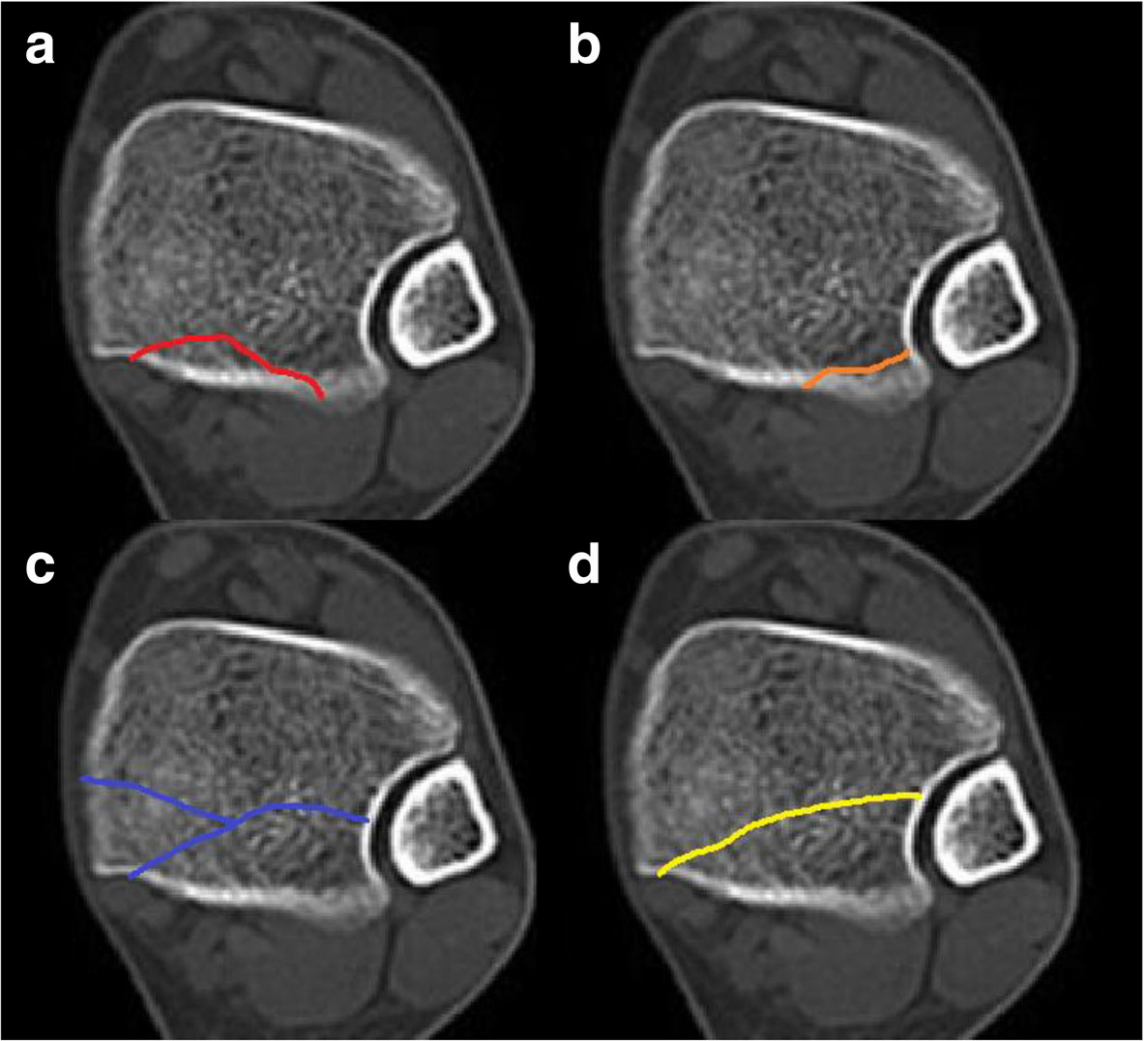
There are four types of Bartoníček’s classification scheme: **a** (type 1): extraincisural fragment with an intact fibular notch; **b** (type 2): posterolateral fragment extending into the fibular notch; **c** (type 3): posteromedial two-part fragment involving the medial malleolus; **d** (type 4): large posterolateral triangular fragment (involving more than one-third of the notch)

Imaging parameters were as follows: 64-detector CT scanner, section thickness, 0.625 mm; tube voltage, 120 kVp; pitch, 1.375; matrix, 512 × 512. Data were saved in DICOM 3.0 format (.dcm) and available in Picture Archiving and Communication System. All the damaged bony structure of the ankle joint was recorded and the PMFs were classified according to Bartoníček’s classification scheme [9]. This is a pathoanatomy-oriented classification based on CT examination that takes into account the size, shape and location of the fragment, stability of the tibiotalar joint and integrity of the fibular notch (Fig. 1). The incidence of a “communication line”, defined as a fracture line progressing from the spiral tibial fracture to the PMF [5], was also recorded.

### 3-D reconstruction and implant simulation

**Fig. 2:**
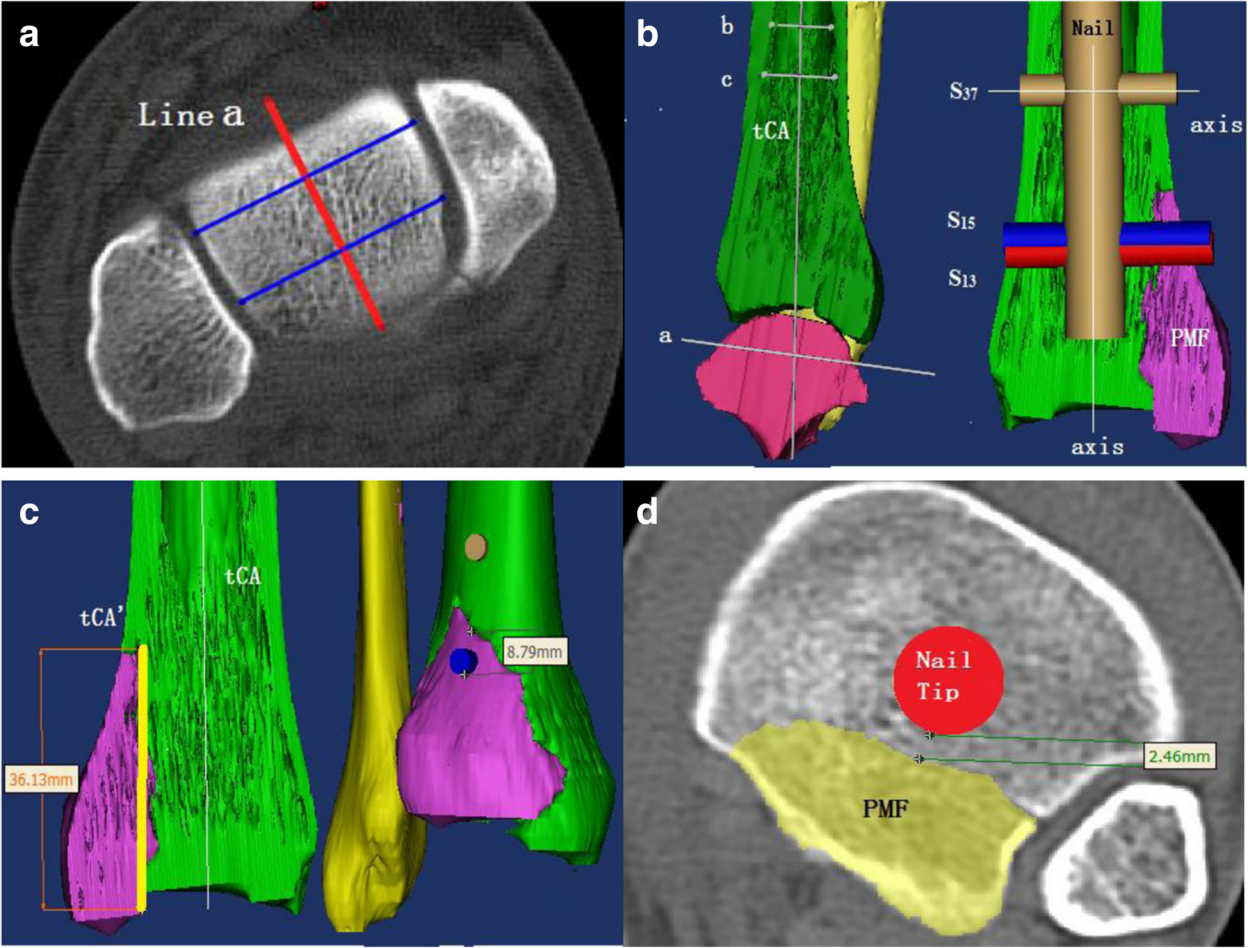
**a** “Line a” is the projection line of the centers of the talar dome. **b** After the central sagittal plane (CSP) is determined, on this plane the tIN and three AP locking screws (S13, S15 and S37) are simulated. The relationship between AP locking screws and the PMF is demonstrated. **c** Measurements on reconstructed 3D model: the fragment height (FH) and the minimal distance from distal AP locking screw tip to the PMF (DSF). **d** Measurements on axial scan: minimal distance from the nail tip to the PMF (DNF)

3-D models were reconstructed with the program Mimics version 19 (Materialise NV Inc., Leuven, Belgium). The model consisted of two components: the lower leg and the simulated vIM nail. After reconstruction of the leg, the simulated vIM nail was devised step by step with a reproducible method. First, as the ideal position of the tibial nail axis is across the center of the ankle, this point and its sagittal plane projection were established on axial scan according to Cinotti’s method [10]. This method is precise [11] and easy to perform by identifying the center of the talar dome originating from the connection of 2 points located in the middle of its anterior and posterior region. The middle points of the talar dome at its anterior and posterior edges were identified with the gauge tool and connected by “line a”; this line is the projection of the center of the ankle in the sagittal plane and represents the central line of the talar dome (Fig. 2a). Second, the geometrical center point of the tibial canal was identified with the area gauge tool of the software. Then, the central sagittal plane of the distal tibia and ankle joint could be determined by connecting “line a” and the aforementioned tibial canal center point. Third, on the central sagittal plane, the tibial canal axis (tCA) was identified using two bisection points of the distal shaft of the tibia described by Yao (Fig. 2b) [12]. The geometrical center point of the tibial canal was also in the tCA which was also the central axis of the vIM nail. On the same plane, the axis of the distal AP locking screws was perpendicular to the tCA (Fig. 2c). Finally, the vIM nail and the distal AP locking screws were reconstructed around their corresponding axis. In this study, the vIM nail was straight, with a diameter of 10 mm and 5 mm for the AP locking screws.

As a rule, the nail tip was positioned just above the distal tibial physeal scar, which is a discrete anatomic landmark at the distal tibia. Three simulated AP locking screws were set at the distal end of the nail. In the reconstructed tibial nail system, each screw was on the central sagittal plane (CSP) and perpendicular to the vIM nail. The distance from the nail tip to the axis of each of these AP locking screws was 13 mm (Screw_13_, S_13_), 15 mm (Screw_15_, S_15_) and 37 mm (Screw_37_, S_37_) to mimic the designs of two widely available tibial nails (TRIGEN META-NAIL Tibial Nail, Smith & Nephew Inc., Massachusetts, USA, and ETN, Depuy Synthes Inc., Zuchwil, Switzerland).

### Radiographic measurements

With the gauge tool Mimics, measurements were conducted separately by two orthopedic surgeons and agreement was reached by consensus. The research method developed by Yao and Haraguchi et al. [12, 13] was applied in the current study. The measured parameters included the height of the PMF (HP), the fragment area ratio (FAR), the minimal distance from nail tip to the PMF (DNP) and the distance from the distal AP locking screw to the PMF (DSP).

### The height of the PMF (HP)

After identification of the tCA, another line (tCA’) was drawn in parallel through the apex of the PMF (Fig. 2c). The HP was defined as the greatest distance from the apex of the fragment to the point where the tCA’ crossed the articular surface of the ankle.

### The FAR

At the level of the tibial plafond, the posterior fragment area (s) and the remaining cross-sectional area of the tibia (S) were measured. The ratio of the fragment area to the total cross-sectional area of the tibial plafond was calculated using (FAR = s / [s + S]).

### The distance from the distal AP locking screws to the PMF (DSP)

The distance from the distal edge of each locking screw to the PMF was measured (Fig. 2c). If the locking screw completely or partially penetrated the PMF, the measured distance was defined as negative and the condition as PMF violation. When the locking screw avoided the PMF, the distance was defined as positive and the condition was defined as fragment spared.

### The distance from the nail tip to the PMF (DNP)

On the axial plane, the minimal distance from tip of the nail to the PMF was measured (Fig. 2d). When there was no violation from the nail tip to the PMF, the distance was defined as positive. When the nail tip violated the PMF, the measurement was defined as negative.

### Interobserver reliability

Two senior orthopedic trauma surgeons analyzed all the CT scans in separate sessions and were blinded to each other’s measurements. The interobserver reliability and 95% CI were excellent for all variables: 0.967 (95% CI, 0.957 to 0.975) for the HP, 0.974 (95% CI, 0.965 to 0.983) for the FAR, 0.967 (95% CI, 0.954 to 0.976) for the DNP, 0.971 (95% CI, 0.962 to 0.983) for the DSP and 0.957 (95% CI, 0.945 to 0.983) for the incidence of secondary fracture line.

### Surgical technique

The patient was supine and fitted with an inflated tourniquet. The subpatellar approach was preferred, and the tibial canal was opened. The fracture was closely reduced by longitudinal manual distraction and maintained with reduction forceps. With the help of the reducer and the Poller screw technique, the guide wire was inserted precisely in the center of the ankle joint in the anteroposterior and lateral view. After gauging and reaming, the tibial nail was inserted with the tip located just above the top of the distal physeal scar in every case, and a lateral radiograph was taken to detect any displacement of the PMF. With a SURESHOT Targeting System (Smith & Nephew Inc., Massachusetts, USA), the most distal AP locking screw was inserted first. The drilling process was monitored under lateral radiography, and any noticeable displacement of the PMF terminated drilling. The PMF was percutaneously reduced with forceps and stabilized with one or two posteroanterior cannulated screws. After confirmation of the PMF reduction, the locking process continued.

### Statistical analysis

The summary measurements were presented as the mean and standard deviation, proportions or as median with Q25 and Q75. Comparisons between the types of posterior malleolar fractures in the incidence of PMF violation were conducted with *t* tests for continuous variables and chi square tests for categorical variables. Two-tailed *p* values of < 0.05 were considered significant.

Variables with *p* values of < 0.2 were manually entered into the multiple logistic regression analysis to model the sparing of the PMF. A variable remained in the model when its *p* value was < 0.05 or when it had a significant impact on the − 2 log likelihood value. The logistic regression model results were presented as ORs with 95% CIs.

Receiver operating characteristic analyses were performed for continuous variables to test for diagnostic accuracy and determine optimal thresholds. Variables with an area under the ROC curve of > 0.80 were considered adequate for investigation [14]. A sensitivity of > 90% was used as the criterion for an optimal threshold.

## Results

Fifty-five patients were included in this study with mean age of 45.18 years (range, 24 to 81 years), and 33 of them were male (60%). The measured FAR and HP of the PMF are listed in Table 1. The DNP was 4.29 mm [range, 1.5–8.54 mm] and all the measurements were positive, which indicated no PMF penetration from the vIM nail tip. Among the three AP locking screws, the S_13_ entailed the highest probability of PMF violation, followed by the S_15_ and S_37._

**Table 1:** Patient demographics and fracture characteristics

Clinical characteristics	n
FAR *(%)*^a^	19.06 ± 8.6
HP *(mm)*	33.38 ± 10.06
Incidence of displaced PMF *(%)*	25.45
Incidence of a “connection line” *(%)*	78.18
Mean displacement of the PMF *(mm)*^b^	1.50 (1.03–1.83)
Incidence of PMF violation *(%)*	
S_13_	50.91
S_15_	43.64
S_37_	1.82
Distance from the nail tip to the PMF *(mm)*	4.29 (1.5–8.54)
Distance from distal AP locking screws to the violated PMF *(mm)*	
S_13_	−7.41 (−13.07 to −5.17)
S_15_	−6.34 (−10.53 to − 5.73)
S_37_	− 1.18

Posterior malleolar fragment (PMF), height of posterior malleolar fragment (HP), fragment area ratio (FAR), anteroposterior (AP)

^a^The values are given as the mean and the standard deviation. ^b^The values are given as the median with Q25 and Q75

According to Bartoníček’s classification, no case fit the description of type 1, and the entire 55 cases could be divided into types 2 to 4. Among those three types, there were significant differences in the incidence of the secondary fracture line (*p =* 0.004), FAR (*p* < 0.001), HP (*p* = 0.0013) and the risk of PMF violation (*p =* 0.004 for the S_13_, and *p =* 0.015 for the S_15_) (Table 2).

**Table 2:** Comparison of demographic and fracture morphology between three types of Bartoníček’s classification

	Bartoníček’s classification			Coefficient value	*p-value*
	Type 2	Type 3	Type 4		
No. of patients	9	7	39		
Age *(yr)*	49.33 ± 14.54	43.00 ± 21.51	44.61 ± 12.07	F = 0.522	0.596
Sex^a^				χ^2^ = 4.105	0.128
Female	7	2	24		
Male	2	5	15		
Secondary fracture line				χ^2^ = 10.878	0.004
Negative	4	4	4		
Positive	5	3	35		
FAR *(%)*	0.05 (0.04–0.09)	0.16 (0.14–0.30)	0.24 (0.19–0.26)	H = 20.585	< 0.001
HP *(mm)*	26.37 (21.51–28.18)	30.98 (26.30–39.40)	33.77 (29.99–39.76)	H = 13.258	0.0013
Incidence of PMF violation					
S_13_				χ^2^ = 11.205	0.004
PMF spared	9	3	15		
PMF disrupted	0	4	24		
S_15_				χ^2^ = 8.414	0.015
PMF spared	9	3	19		
PMF disrupted	0	4	20		

Height of posterior malleolar fragment (HP), fragment area ratio (FAR), posterior malleolar fragment (PMF)

^a^The values are given as the number of patients

Univariate analysis revealed that for the S_13_, the difference between the spared and violated PMF was significant in FAR (*p* = 0.004), HP (*p* < 0.001) and Bartoníček’s classification distribution (*p* = 0.004). There were also significant differences between the two groups in FAR (*p* = 0.003), HP (*p* < 0.001) and Bartoníček’s classification distribution (*p* = 0.015) for the S_15_ (Table 3). Multiple logistic regression analysis showed the HP as an independent factor for predicting spared PMF for the S_13_ (OR 1.18, 95% CI 1.06–1.32, *p* = 0.002) and the S_15_ (OR 1.15, 95% CI 1.05–1.27, *p* = 0.005).

**Table 3:** Univariate analysis results for factors related to posterior malleolar fragment violation of S_13_ and S_15_

Clinical features	S_13_		*p-value*	S_15_		*p-value*
	PMF spared	PMF disrupted		PMF spared	PMF disrupted	
FAR *(%)*^a^	0.14 (0.07–0.24)	0.24 (0.19–0.26)	0.004	0.14 (0.08–0.24)	0.24 (0.19–0.27)	0.003
HP *(mm)*	27.23 (23.6–0.71)	37.22 (33.12–41.03)	< 0.001	28.16 (24.69–30.98)	37.22 (33.17–41.24)	< 0.001
Bartoníček’s classification^b^			0.004			0.015
Type 2	9	0		9	9	
Type 3	3	4		3	4	
Type 4	15	24		19	20	

Posterior malleolar fragment (PMF), height of posterior malleolar fragment (HP), fragment area ratio (FAR)

^a^The values are given as the median with Q25 and Q75. ^b^The values are given as the number of patients

For the S_13_ and the S_15_, the only continuous radiographic variable with an adequate AUC value in the ROC was the HP (0.909 for S_13_ and 0.882 for S_15_). For the S_13_, the HP cutoff value of 31.2 mm was 88.89% sensitive and 88.89% specific for spared PMF, corresponding to a positive and negative predictive value of 85.71% and 88.89%, respectively. An HP cutoff value of 31.2 mm was 88.89% sensitive and 88.89% specific for spared PMF, corresponding to a positive and negative predictive value of 85.71% and 88.89%, respectively. For the S15, this cutoff was 80.65% sensitive and 87.50% specific for PMF violation, corresponding to a positive and negative predictive value of 89.29% and 77.78%, respectively.

**Fig. 3:**
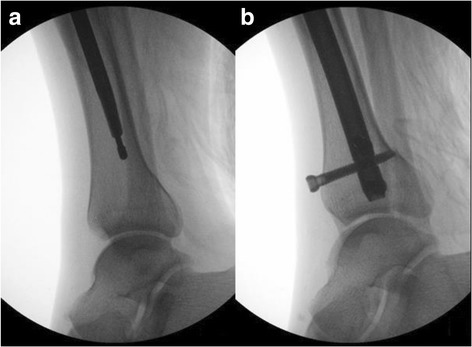
**a** Demonstrating a non-displaced PMF before intramedullary nail insertion. **b** The penetration of distal tibial physeal scar from nail tip resulted in a secondary displacement of the PMF from distal AP locking screw

From the same group, tibial IM nails (TRIGEN META-NAIL Tibial Nail, Smith & Nephew Inc., Massachusetts, USA) were applied to 35 cases with no significant initial displacement of the PMF. The HP in 18 patients was less than 31.2 mm. Of the 18 cases, 6 patients experienced distal migration of the nail and physeal scar penetration during nail insertion. Five of 6 had PMF secondary displacement caused by S_15_ screws (Fig. 3). The remaining 12 cases whose IM nails were properly positioned had no secondary displacement. Among the 17 cases with PMF height exceeding 31.2 mm, 3 cases had distal physeal scar penetration. Fourteen cases had secondary displacement of PMF caused by an S_15_ screw, including three physeal scar penetrating cases.

## Discussion

After Lauge-Hansen’s discussion [1], Weber’s series including 14 similar cases evoked interest in this topic [3]. Then, other authors conducted further studies investigating the injury mechanism, radiographic features, and associated injuries resulting from the treatment protocol [15–17]. The greatest improvement was made in summarizing the radiographic characteristics of this specific lower injury. When plain radiography was the primary tool for fracture assessment, Werken stated that a concomitant posterior malleolar fragment was actually an isolated fracture [2]. However, Hou’s study revealed that the incidence of a “connection line” in concomitant tibial shaft fracture and ipsilateral ankle injury was as high as 92.71% [5]. In agreement with that conclusion, our study verified the high occurrence of this distinct radiographic sign (78.18%). When evaluated by CT scan only, our results confirmed that the “connection line” was a reliable indicator of concomitant posterior malleolar fracture and distal tibial spiral fracture.

The optimal treatment has been a topic of debate in recent years. One source of disagreement is the optimal implantation selection. Intramedullary nails and minimal invasive plating techniques can both be utilized to fix a tibial fracture. Generally, IM nails with locking screws can provide sufficient fixation stability with minimal soft tissue dissection [18, 19]. Furthermore, the fixation stability of distal tibial shaft fractures has been substantially improved by multiple-level/multiple-direction locking options and the Poller screw technique [20, 21]. Recently, most authors have preferred the IM nail rather than plating to treat this injury [2, 5, 6, 16, 22–24].

Another subject of interest has been how to treat PMF. It is clear that a considerably displaced PMF requires reduction and fixation to reconstruct the tibial plafond articular surface to congruency [25]. However, the present study revealed that in most cases, the PMF was non-displaced (74.55%), and the mean FAR was 19.06 ± 8.6%, much smaller than the proposed critical size for a posterior malleolar fracture fragment, which is one-quarter to one-third of the articular surface [26]. From the standpoint of rehabilitation, the fixed PMF might provide the theoretical benefit of allowing early active/passive ankle flexion exercise and even early partial weight bearing. However, biomechanical study has revealed that in normal ankles, the contact stresses are centrally located in plantar flexion and move to an anterior location as the ankle moves toward dorsiflexion. Therefore, a large area of the cartilage at the periphery of the joint is not loaded during the motion cycle. Under physiological conditions, the posterior articular surface of the ankle bears little load during the range of motion [25]. Thus, when a non-displaced PMF is left unfixed, the non-weight-bearing exercise of ROM would be unlikely to cause secondary displacement.

To gain a better holding strength on the distal tibial segment, deeper insertion of the IM nail was advocated. However, when nailing a tibial shaft fracture concurrent with ipsilateral posterior malleolar fracture, the PMF is within the pathway of the nail and distal AP screws. An increased depth of the nail would exaggerate the proximity between the nail/screw and the PMF. As a result, the reaming of the canal or the drilling of the AP locking screw might cause secondary displacement of the PMF. Therefore, the only indication for PMF fixation might be the necessity of eliminating the risk of secondary displacement of this intra-articular fracture during tibial nailing.

To quantify the actual risk of secondary displacement of the PMF during nail insertion and screw locking, we devised a simulated operation, and the results were confirmed by actual operations. In this study, the distal tibial physeal scar was used as the reference for nail insertion depth to gain the best holding strength because this structure is consistent in almost every case [8, 27]. It is also a durable bone plate that can withstand further advancement of the nail and provide obvious biomechanical benefits to fixation stability.

After the standardization of the IM nail insertion depth, a vIM nail with multiple AP locking screw options was simulated. The DNP measurement revealed that setting the advancement limit of the nail tip provided a safe space between the nail and the PMF (4.29 mm [1.5–8.54]). Therefore, the nail itself would not greatly exacerbate the posterior malleolar fracture displacement when the nail tip depth was properly determined. Subsequent study revealed that secondary displacement was almost caused by distal AP locking screws S_13_ and S_15_. Further comparison between the violated and spared PMF groups revealed that the FAR and HP were significantly different (Table 3). This result confirmed that the morphological features of the PMF would significantly influence the risk of PMF violation.

As a morphological classification, Bartoníček’s classification highly correlated with the risk of PMF violation during nailing. For S_13_ and S_15_, there were significant differences among the three types in the rate of PMF violation (*p* = 0.004 and *p* = 0.015), and the highest group was type 4 (61.54% and 48.72%), followed by type 3. This result suggested that the surgeon should be alerted to the risk of PMF secondary displacement when the cross-section area and height of the PMF are large. To further quantify the risk factors, univariate analysis and multiple logistic regression analysis identified HP as the single significant factor relating to the violation of PMF by S_13_ and S_15_. ROC analysis revealed that HP < 31.2 mm could be a reliable independent protective factor for PMF violation. According to the study design, this parameter was established using the distal tibial physeal scar as the consistent nail depth limitation. When the nail stopped proximally to this reference, the risk of PMF violation was reduced accordingly, while the mean distance measured from S_13_ and S_15_ to the edge of the violated PMF was − 7.4 mm (from − 2.32 to − 24.52) and − 6.34 mm (from − 2.08 to − 22), respectively. It can be postulated that withdrawal of the inserted vIM nail by 1.5 cm could significantly reduce the risk of PMF violation for both S_13_ (from 50.91% to 9.09%) and S_15_ (from 43.64% to 5.45%). The threshold of the HP is a very precise number and we believe it is unnecessary to normalize this figure according to patient’s height or body build. The risk of PMF secondary displacement is mainly determined by the fragment’s own anatomic features. Furthermore, there is no evidence that height or body build could significantly alter the position of the distal tibial physeal scar. Although the length of the tibial nail is significantly influenced by the height variance among patients, the distal locking screw arrangement in a tibial nail of any size is standardized.

The results of this simulation study were subsequently validated in the actual patients. Of the 55 patients, 35 patients had non-displaced PMF; therefore, their intraoperative and postoperative radiography were investigated. When the height of the PMF was less than 31.2 mm, avoiding distal tibial physeal scar penetration by the nail tip could act as a protective factor of the PMF from distal AP locking screw violation (S_13_ and S_15_). However, this effect was not significant when the HF exceeded 31.2 mm. For those cases, to eliminate the risk of PMF secondary displacement, advanced PMF fixation by a cannulated screw before nail insertion is suggested.

We acknowledge the inherent limitations of the current study. Foremost, we used a relatively small sample size; however, we believe the use of non-paired objects of study provided sufficient sample heterogeneity. Furthermore, the relatively narrow ranges of our confidence intervals suggest that the sample size was sufficient for the observed variability. Second, the simulated nail fixation process was devised under idealized conditions that might not fully reflect reality; thus, the conclusion of this study could not cover all the fractures, especially when a PMF is more complex, such as with large size, substantial displacement or incarcerated fragments. However, this result was justified by our retrospective analysis of the authentic operation radiographic data. Our study provided concrete support for Guo’s suggestion [8] that the traditional indication can be used to guide the treatment of a PMF. Third, our vIM nail does not cover all types of authentic tibial nails. Actually, it is impractical to make the virtual model represent all nail designs. To the best of our knowledge, the two prototypes are among the most widely used tibial intramedullary nails and both are characterized by an extremely distally located locking screw design. Therefore, we believe this virtual model has certain representativeness.

## Conclusion

In this paper, the potency of Bartoníček’s classification in directing the treatment of a PMF combined with a distal tibial spiral fracture was confirmed. We further found three key factors underpinning the risk of PMF secondary displacement: (1) the height of the PMF, (2) the space relationship between the nail tip and the distal tibial physeal scar and (3) the position of the distal anteroposterior locking screw in the tibial intramedullary nail. Among all the factors, the HP and the physeal scar were consistent variants and out of the surgeon’s control. Identification of them could help the treating surgeon eliminate the risk of PMF secondary displacement by using a simple mathematical calculation. Other than preliminary PMF fixation, the surgeon could either draw the tibial nail (with a distal locking screw arrangement similar to the vIM nail) back a little to maintain a safe distance between the nail tip and the physeal scar, or change to another tibial nail with a more proximal tibial anteroposterior locking screw design.

## Abbreviations

AP: anteroposterior
CI: confidence interval
DNP: distance from the nail tip to the PMF
DSP: distance from the distal AP locking screw to the PMF
FAR: fragment area ratio
HP: height of the PMF
OR: odds ratio
PMF: posterior malleolar fragment
S_13_: Screw_13_
S_15_: Screw_15_
S_37_: Screw_37_
tCA: tibial canal axis
vIM: nail virtual tibial intramedullary nail
